# PLK1 inhibition impairs erythroid differentiation

**DOI:** 10.3389/fcell.2024.1516704

**Published:** 2024-12-23

**Authors:** Peijun Jia, Yan Li, Lulu Duan, Jingxin Zhang, Yuanlin Xu, Huan Zhang, Chenghui Wang, Yaqian Gong, Ying Zhang, Longzhen Zhao, Yumin Huang, Shijie Zhang

**Affiliations:** ^1^ School of Life Sciences, Zhengzhou University, Zhengzhou, China; ^2^ Department of Internal Medicine, The Affiliated Cancer Hospital of Zhengzhou University & Henan Cancer Hospital, Zhengzhou, China; ^3^ Department of Hematology, The First Affiliated Hospital of Zhengzhou University, Zhengzhou, China

**Keywords:** plk1, erythropoiesis, anemia, apoptosis, cell cycle

## Abstract

Polo-like kinase 1 (PLK1), a key regulator of the G2/M phase in mitosis, is frequently overexpressed in numerous tumors. Although PLK1 inhibitors have emerged as promising therapeutic agents for cancer, their use has been linked to significant anemia in a subset of patients, yet the underlying mechanisms remain poorly understood. In this study, we utilized an *in vitro* human umbilical cord blood-derived CD34^+^ cell-based erythroid differentiation system, alongside a murine model, to investigate the impact of PLK1 inhibitors on erythropoiesis. Our results indicate that PLK1 inhibitors, specifically GSK461364 and BI6727, significantly suppress the proliferation of erythroid cells, resulting in G2/M phase cell cycle arrest, increased apoptosis in erythroid cells, and the formation of abnormally nucleated late-stage erythroblasts. *In vivo*, administration of PLK1 inhibitors in mice induced severe anemia, as evidenced by a marked reduction in red blood cells and hemoglobin levels. More specifically, PLK1 inhibition impaired the differentiation and erythroid commitment of hematopoietic stem cells in the bone marrow, resulting in abnormal accumulation of BFU-E cells and reduced proliferation and differentiation of CFU-E, and a decrease in the number of terminal erythrocytes. Mechanistically, PLK1 inhibitors primarily induce apoptosis in erythroid cells by reducing Mitochondrial membrane potential and arresting the cell cycle at the G2/M phase. Overall, our findings underscore the critical role of PLK1 in erythropoiesis and shed light on the mechanisms underlying PLK1 inhibitor-induced anemia, providing essential guidance for developing strategies to prevent and manage anemia in clinical applications of PLK1-targeted therapies.

## Introduction

Polo-like kinase 1 (PLK1), highly expressed in various primary tumor tissues including glioblastomas, thyroid cancer, head and neck squamous cell carcinoma, and acute myeloid leukemia, has become a promising target for cancer therapy due to its role in promoting tumorigenesis (De Martino et al., 2018; Hagege et al., 2021; Lerner et al., 2015; Xia et al., 2023). Preclinical studies show that blocking PLK1 pathways effectively suppresses tumor cell growth and induces apoptosis. Several PLK1 inhibitors, including Volasertib (BI6727), BI2536, NMS-1286937, GSK461364, MK-1496, TKM-080301, Rigosertib, TAK-960, and CFI-400945, have advanced to clinical trials, paving the way for further exploration of their cellular functions (Goroshchuk et al., 2019; Lian et al., 2018; Pajtler et al., 2017; Su et al., 2022; Van den Bossche et al., 2016). Despite the widespread application of PLK1-targeted therapies in cancer trials, anemia remains a common complication (Awada et al., 2015; Cortes et al., 2021; Lin et al., 2014). Anemia can substantially compromise the therapeutic efficacy of PLK1 inhibitors in oncology. The reduction in red blood cell count results in diminished oxygen transport to tissues, which not only adversely impacts patients' overall physiological status and quality of life but also attenuates their responsiveness to anti-cancer therapies.

Anemia, often associated with impaired erythropoiesis, is a frequent issue in cancer treatment (Bohlius et al., 2019). Erythropoiesis is a complex process encompassing early erythropoiesis, terminal erythroid differentiation, and reticulocyte maturation. During early erythropoiesis, hematopoietic stem cells differentiate into burst-forming unit-erythroid (BFU-E) and colony-forming unit-erythroid (CFU-E) cells (Yang et al., 2024). Terminal differentiation progresses through stages of proerythroblasts, basophilic erythroblasts, polychromatic erythroblasts, and orthochromatic erythroblasts (Wang et al., 2021). Disruption at any stage can result in ineffective erythropoiesis and anemia (Ginzburg et al., 2023). However, the specific role of PLK1 in erythroid development remains largely unexplored.

Among the PLKs family, PLK1 is the most extensively studied member due to its critical role in cell cycle progression, particularly at the G2/M checkpoint (Didier et al., 2008). Activated by binding to phosphorylated proteins, PLK1 works in concert with cyclin-dependent kinase 1 (CDK1), cyclin B1, and Aurora A or Aurora B to regulate key mitotic events (Strebhardt, 2010). These functions include centrosome maturation and spindle assembly, positioning PLK1 as a master regulator of mitotic entry and progression. During mitosis, PLK1 is dynamically localized, starting at the centrosomes during early mitosis, migrating to the spindle poles, and accumulating at the midbody during cytokinesis (Colicino and Hehnly, 2018). Expression of PLK1 is tightly regulated, peaking in M phase and sharply declining upon mitotic exited (Chiappa et al., 2022).

In this study, we employed two PLK1-specific inhibitors, GSK461364 and BI6727, to investigate the impact of PLK1 on erythropoiesis in both human and murine models. Our results demonstrated that PLK1 inhibition disrupts erythroblast proliferation and differentiation, leading to apoptosis and cell cycle arrest. *In vivo* studies further confirmed that PLK1 inhibitors cause severe anemia in mice, disrupting bone marrow (BM) hematopoietic stem cell homeostasis. Moreover, PLK1 inhibition block erythroid differentiation and induce both apoptosis and cell cycle arrest at the G2/M phase in erythroblasts. Elucidating the mechanisms by which PLK1 inhibition causes anemia provides valuable insights for the prevention and treatment of anemia in clinical settings, while also offering new strategies for the development of cancer therapeutics and combinatory treatments.

## Materials and methods

### Animal experiments

The mouse experiments were approved by the Animal Ethics Committee of the Institute of Zhengzhou University (ZZUIRB 2023–243). Wild-type C57BL/6 mice (8 weeks old, weighing 20 ± 2 g) were purchased from Charles River. The mice were randomly assigned to either the control group or the BI6727 treatment group. BI6727 was administered at a dose of 25 mg/kg via intraperitoneal injection once a week for 2 weeks. The vehicle used was a mixture of PEG 300, Tween 80, and saline. Control group mice received only the vehicle. Mice were euthanized on Day 14 for further analysis.

### Cell culture

CD34^+^ cells were isolated from human cord blood using magnetic activated cell sorting (MACS) per the manufacturer’s instructions, with a purity of 95% to 98%. Cells were cultured in Iscove’s Modified Dulbecco’s Medium (IMDM) supplemented with 8% fetal bovine serum (FBS), insulin (10 μg/mL), heparin (3 IU/mL), SCF (10 μg/mL), IL-3 (1 μg/mL), EPO (3 IU/mL) and penicillin-streptomycin (1%). Cultures were maintained at 37°Cin 5% CO_2_. The culture process was divided into four phases. From Day 0 to Day 7, cells were cultured in full medium at 1 × 10^5^/mL. IL-3 was removed from day 7 to day 11, SCF and IL-3 were excluded from day 11 to day 15, and EPO was omitted in the final phase. K562 cell line were cultured in 1,640 medium containing 15% FBS.

### Flow cytometry analysis

For mouse BM, cells were flushed with PBS containing 2% FBS and 2 mM EDTA, then filtered through a cell strainer into a centrifuge tube. Lineage-positive cells were depleted, and flow cytometry was conducted to analyze hematopoietic stem and progenitor cell (HSPC) development and erythropoiesis, following established methods. *In vitro* erythroid cells were collected from culture and analyzed for surface markers including glycophorin A (GPA), IL-3R, CD34, CD36 and CD105, along with Hoechst 33,342 to assess early and terminal erythroid differentiation and enucleation. Antibody protocols followed previous publications (Xu et al., 2022; Zhang et al., 2021). Apoptosis was assessed using an Annexin V kit (eBioscience, #88–8,103-74).

### Cell cycle analysis

Cell cycle analysis was performed using an EdU staining kit (Invitrogen, C10632) following the manufacturer’s instructions. Briefly, one million cells were cultured with 10 μM EdU for 2 h to incorporate EdU into newly synthesized DNA during active DNA replication. After incubation, the cells were harvested and fixed with 4% paraformaldehyde for 15 min at room temperature. The Click-iT reaction mixture was then prepared and added to the cells, enabling the fluorescent labeling of EdU. After incubation in the dark for 30 min, the cells were washed with PBS, resuspended in buffer, and analyzed by flow cytometry to assess cell cycle progression.

### May-Grunwald Giemsa staining and analysis

Cells were harvested and placed onto glass slides using a cytocentrifuge. The slides were first stained with May-Grunwald solution (Sigma) for 5 min, then transferred to freshly prepared 10% Giemsa solution for 10 min. After staining, the slides were gently rinsed twice with deionized water, air-dried, and sealed with mounting medium. The stained slides were examined using an optical microscope, and images were captured for further analysis. Cell differentiation stages were determined based on cell size using Image-Pro software for image analysis.

### Colony formation assay

For the colony formation assay, *in vitro* cultured cells were plated in triplicate at a density of 200 cells per well in 1 mL of MethoCult H4434 (Stemcell Technologies, #04434) medium for BFU-E colony analysis, and in MethoCult H4330 (Stemcell Technologies, #04330) for CFU-E assays. The plates were incubated at 37°C with 5% CO_2_ in a humidified incubator. Colony counts for CFU-E and BFU-E were performed on days 7 and 14, respectively. Mouse BM cells were also plated in triplicate at a density of 50,000 cells per well in 1 mL of MethoCult M3334 (Stemcell Technologies, #03334) or M3434 (Stemcell Technologies, #03434) (Han et al., 2024). These plates were incubated under identical conditions, with CFU-E and BFU-E colonies counted on days 3 and 7, respectively.

### Examination of blood parameters

Mice blood samples were collected from the suborbital vein and then examined by ADVIA^®^2120i hematology system (Siemens).

### Statistical analysis

All experiments were conducted in a randomized and blinded manner. Flow cytometry data were analyzed using FlowJo software, while ImageJ was employed for analyzing band signal intensities. Statistical analyses were performed with GraphPad Prism 8.0. Data are presented as mean ± SD, with statistical significance set at *p < 0.05, **p < 0.01, ***p < 0.001.

## Results

### Expression of PLK1 during human erythroid differentiation

**FIGURE 1:**
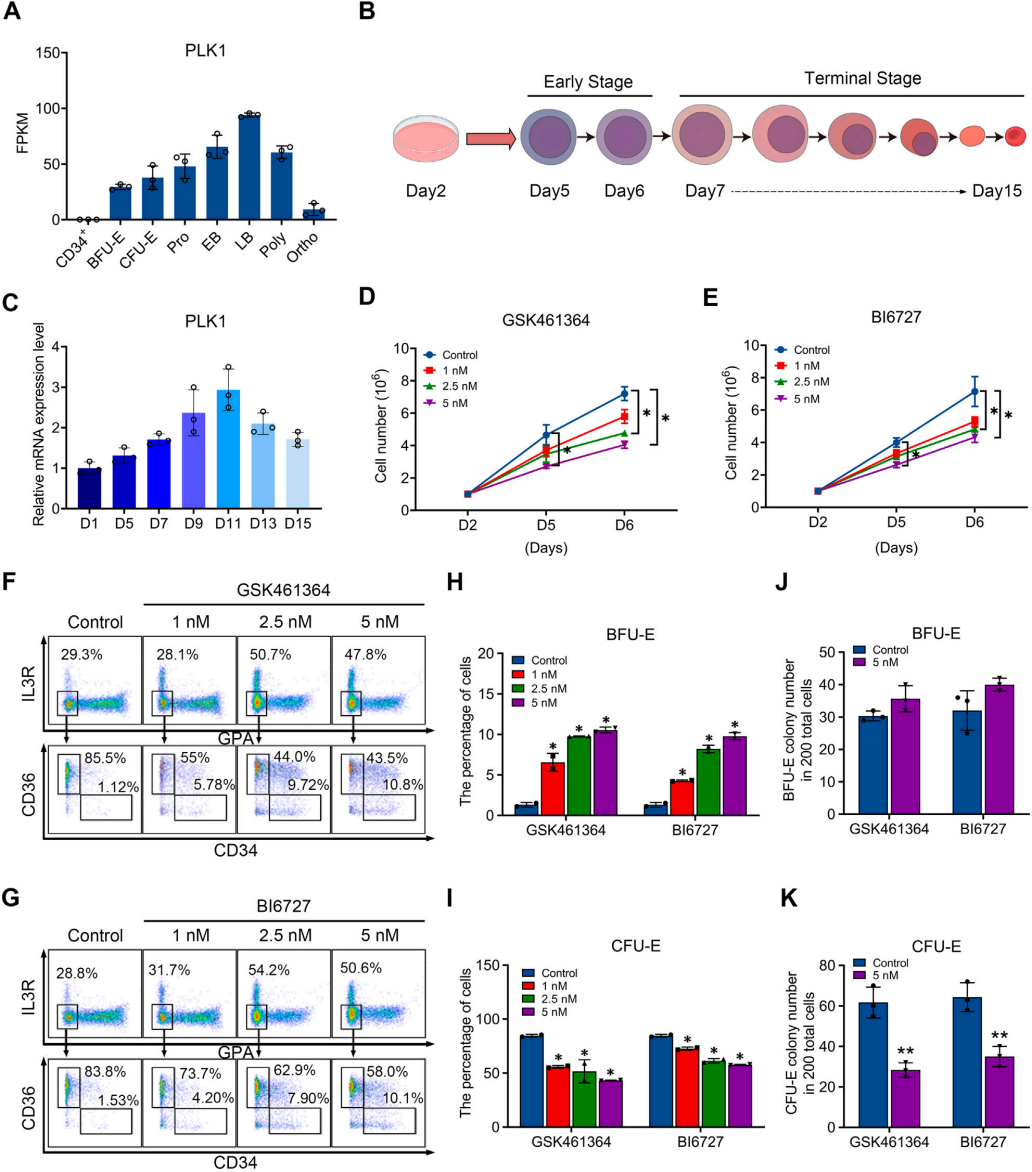
PLK1 downregulation impairs early erythropoiesis. **(A)** RNA-sequencing analysis of PLK1 mRNA expression levels at different developmental stages of erythroblasts cultured from peripheral blood CD34^+^ cells. **(B)** Schematic diagram of erythroid cell differentiation from CD34^+^ cells. **(C)** qRT-PCR analysis of PLK1 mRNA expression. **(D, E)** Growth curves of erythroblasts treated with PBS, GSK461364, and BI6727 over 2, 5, and 6 days. **(F, G)** Representative flow cytometric analysis of human erythroid progenitors treated with PBS or varying concentrations of GSK461364 and BI6727 on day 6. **(H, I)** Proportions of BFU-E and CFU-E cells on day 6 of CD34^+^ cell culture. **(J, K)** Number of BFU-E and CFU-E colonies formed from 200 plated cells cultured on day 6 in the presence of PBS, GSK461364, and BI6727. The experiments were repeated 2–3 times with three technical replicates per condition. *P < 0.05, **P < 0.01.

In order to investigate the role of PLK1 in erythropoiesis, we examined their expression during human erythroid differentiation. Figure 1A shows our RNA-sequencing data of erythroblasts cultured from CD34^+^ cells at distinct stages of development, which reveals abundant expression of PLK1 with distinct expression patterns, with PLK1 upregulated in late-stage erythroblasts. We differentiated cord blood-derived CD34^+^ cells to erythroid cells using a three-step culture erythroid system, as shown in Figure 1B. The expression patterns of PLK1 were further confirmed by quantitative real time polymerase chain reaction (qRT-PCR) analysis (Figure 1C). The constant and abundant expression of PLK1 suggest a potentially important role during human erythropoiesis.

### PLK1 inhibition impairs erythroid progenitor differentiation and proliferation

To test our hypothesis, we utilized two inhibitors to induce PLK1 dysfunction during erythropoiesis. Specifically, we treated CD34^+^ cells in an *in vitro* erythroid induction system with GSK461364 and BI6727, which are selective inhibitors of PLK1, starting on day 2 of culture. By day 6, we observed a significant, dose-dependent reduction in cell proliferation, with GSK461364 demonstrating stronger inhibitory effects than BI6727 at comparable concentrations (Figures 1D, E). Erythroid differentiation, particularly the transition from BFU-E to CFU-E, was assessed using surface markers glycophorin A (GPA), IL3-R, CD34, and CD36. Flow cytometry analysis on day 6 revealed that both inhibitors increased the percentage of BFU-E and reduced the percentage of CFU-E, suggesting that PLK1 inhibition arrested cells at the BFU-E stage and impeded their progression to CFU-E (Figures 1F–I). Furthermore, the inhibitory effect on erythroid progenitor proliferation was corroborated by a increased in the number of BFU-E colonies and a corresponding decrease in CFU-E colonies in PLK1 inhibitor-treated cells compared to controls on days 5 and 6 (Figures 1J, K). These findings indicate that PLK1 inhibition specifically disrupts erythroid progenitor differentiation.

### PLK1 inhibition delays human terminal erythroid differentiation

**FIGURE 2:**
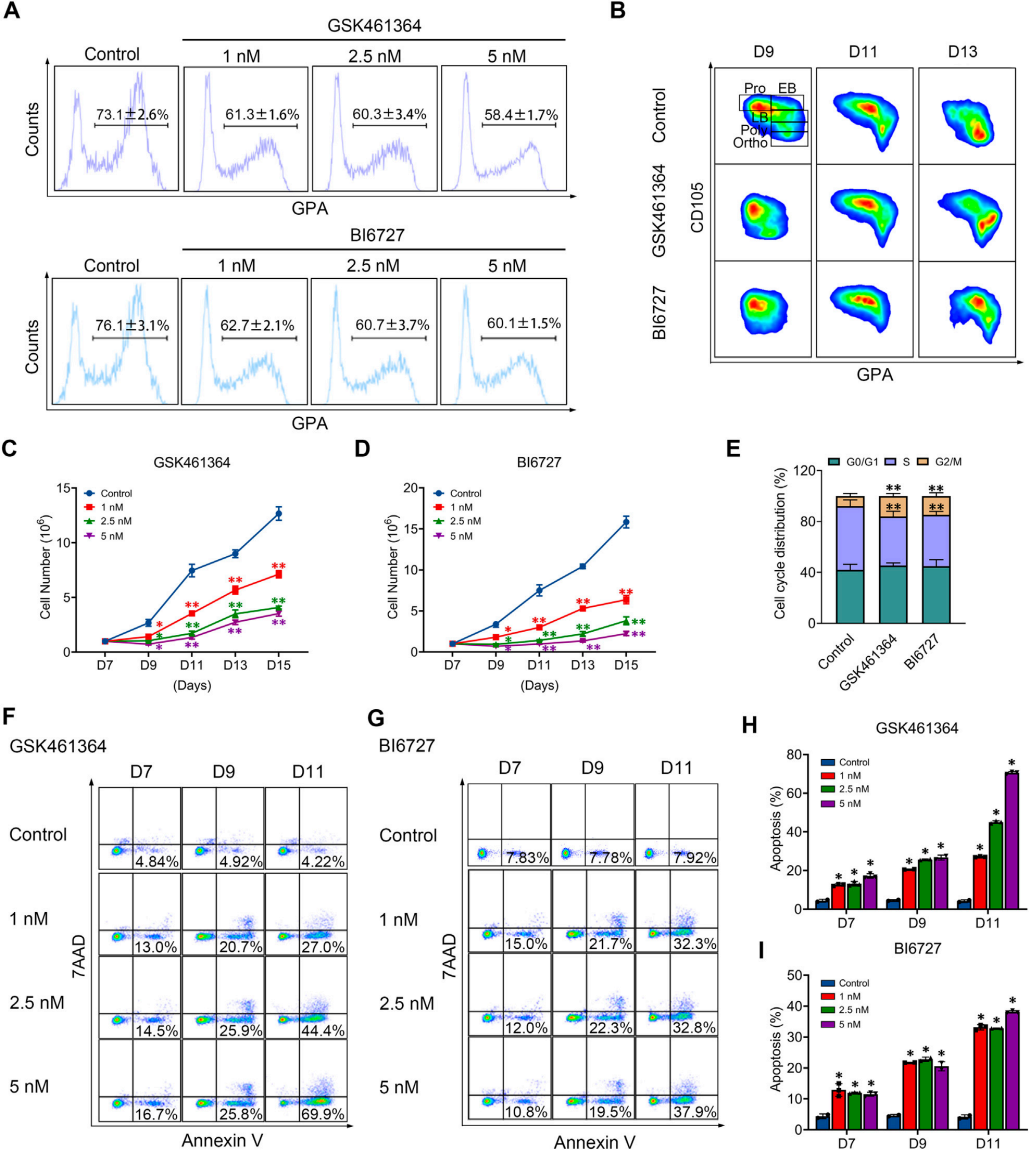
PLK1 downregulation impairs terminal erythropoiesis. **(A)** Flow cytometry analysis of GPA expression in erythroblasts treated with PBS, GSK461364, and BI6727 on day 7. **(B)** Flow cytometry analysis of CD105 and GPA expression in erythroblasts on days 9, 11, and 13. **(C, D)** Growth curves of erythroblasts treated with PBS, GSK461364, and BI6727 over 7, 9, 11, 13, and 15 days. **(E)** Cell cycle distribution analysis in erythroblasts treated with PBS, GSK461364, and BI6727 on day 7. **(F, G)** Flow cytometry profiles showing apoptosis in erythroblasts on days 7, 9 and 11. **(H, I)** Quantitative analysis of apoptosis. The experiments were repeated 2–3 times with three technical replicates per condition. *P < 0.05, **P < 0.01.

We then examined the effects of PLK1 inhibition on terminal erythroid differentiation, the process during which morphologically recognizable proerythroblasts derived from CFU-E cells undergo four to five mitosis to generate enucleated reticulocytes. The differentiation of CFU-E cells to proerythroblasts is characterized by the surface expression of the erythroid specific marker GPA. As shown in Figure 2A, while more than 70% of control cells were GPA positive on day 7, less than 63% of both PLK1 inhibitors treated cells were GPA positive, implying PLK1 inhibition impairs differentiation of CFU-E to proerythroblasts. Differentiation of proerythroblasts to later stage erythroblasts is characterized by increased surface expression of GPA and decreased surface expression of CD105. As shown in Figure 2B, the upregulation of GPA and downregulation of CD105 were significantly delayed in PLK1 inhibition cells compared to control cells, implying delayed terminal erythroid differentiation.

### PLK1 inhibition results in reduced cell growth of late stage erythroblasts

We next examined whether PLK1 inhibition affects the proliferation of erythroid cells. Treatment with GSK461364 and BI6727 significantly impaired cell growth compared with the control group (Figures 2C, D). To explore the mechanisms responsible for impaired cell growth, we first examined the effects of PLK1 on cell cycle progression. Notably, PLK1 inhibition led to cell cycle arrest at the G2/M phase (Figure 2E). Furthermore, we analyzed the effects of PLK1 inhibition on apoptosis through Annexin V staining on days 7, 9, and 11 in both control and PLK1 inhibitor-treated groups. The results revealed that apoptosis became detectable by day 7, with a progressive increase over time, reaching rates of over 50% by day 11 in groups treated with the highest concentrations of GSK461364 and BI6727 (Figures 2F–I).

We further employed GSK461364 and BI6727 in human erythroleukemia K562 cells, selected for their ability to differentiate along the erythroid lineage upon hemin treatment, to verify the role of PLK1 in erythroid development. Both treatments with GSK461364 and BI6727 resulted in a significant reduction in cell numbers compared to control cells between days 3 and 4 post-hemin induction (Supplementary Figures S1A, B). Representative profiles of 7AAD and Annexin V staining from days 1 to 4 in K562 cells treated with GSK461364 and BI6727 following hemin induction are displayed in Supplementary Figures S1C, E. Quantitative analysis revealed a marked increase in apoptosis in PLK1 inhibitor-treated K562 cells on days 3 and 4 (Supplementary Figures S1D, F). These results indicate that PLK1 inhibition leads to reduced cell proliferation and increased apoptosis during erythroid differentiation in K562 cells, highlighting its critical role in erythropoiesis.

### Generation of erythroblasts with abnormal nucleus following PLK1 inhibition

**FIGURE 3:**
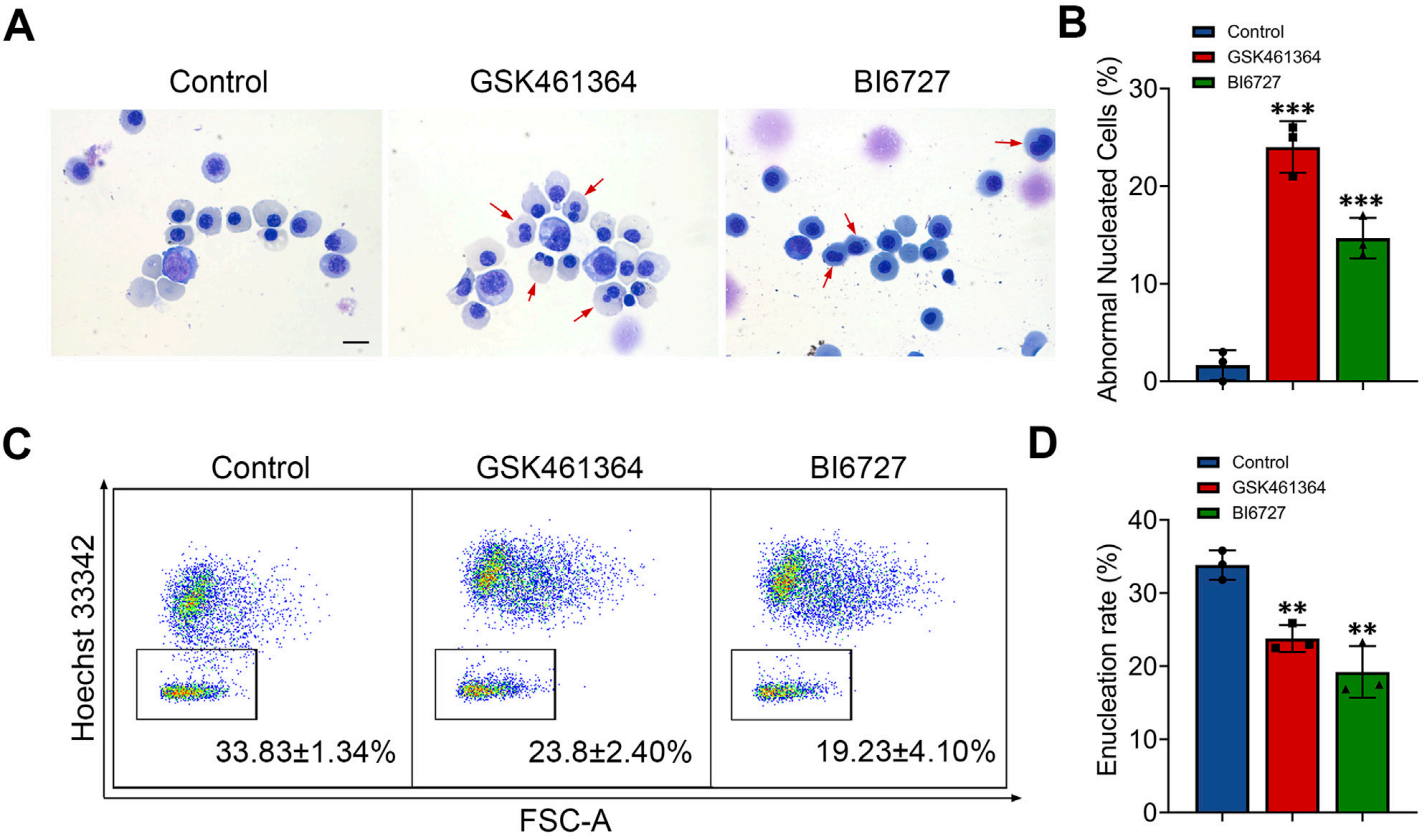
PLK1 inhibition leads to abnormal enucleation. **(A)** Representative cytospin images of erythroblasts cultured for 13 days. Scale bar = 10 μm. **(B)** Quantitative analysis of the proportion of erythroblasts with abnormal nuclei on day 13 from three independent experiments. **(C)** Representative flow cytometry plots showing enucleation efficiency in control and PLK1-inhibited erythroblasts. **(D)** Quantitative analysis of enucleation efficiency from three independent experiments on day 15. The experiments were repeated 3 times with three technical replicates per condition. **P < 0.01, ***P < 0.001.

When examining the effects of PLK1 inhibition on morphology of erythroid cells, we surprisingly found that terminally differentiated erythroblasts exhibited abnormal nuclei following both GSK461364 and BI6727 treatment (Figure 3A). Quantitative analysis of data derived from three independent experiments showed that PLK1 deficiency significantly increased the generation of cells with abnormal nuclei, with approximately 22% and 15% on days 13 (Figure 3B). We used flow cytometry to examine the enucleation status of cells stained with Hoechst 33,342 and found that treatment with both GSK461364 and BI6727 resulted in a significant decrease in enucleation on day 15 (Figures 3C, D).

### PLK1 inhibitor treatment induces anemia in mice

**FIGURE 4:**
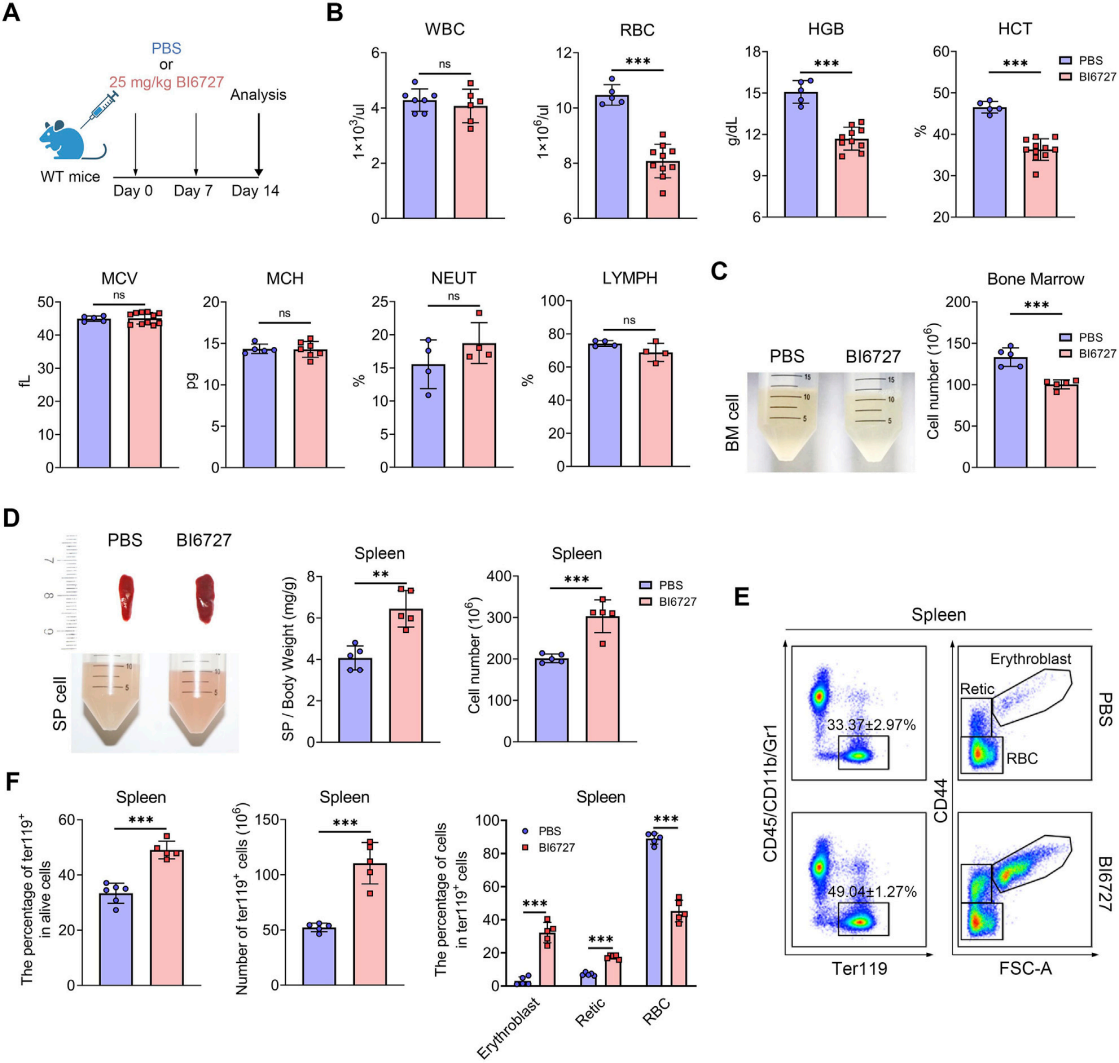
PLK1 inhibition induces anemia in mice. **(A)** Schematic representation of BI6727 injection in mice. **(B)** Complete blood count analysis of short-term BI6727-treated mice. **(C)** Morphological examination of BM suspension. Quantification of total BM cell counts. **(D)** Morphological examination of spleens, and spleen cell suspension. Quantification of spleen/body weight and spleen cell counts. **(E)** Representative flow cytometry plot of splenic erythropoiesis in mice treated with PBS and BI6727. **(F)** Quantitative analysis of Ter119^+^ cells, erythroblasts, reticulocytes, and RBCs in mice spleen samples. N = 5. **P < 0.01, ***P < 0.001. N.S.: no statistic different.

To further explore the role of PLK1 in erythroid development, we administered BI6727 injections to healthy C57BL/6 mice at a dose of 25 mg/kg, given once weekly for two consecutive weeks (Figure 4A). This treatment regimen resulted in no mortality but successfully induced a distinct hematological phenotype. Notably, PLK1 inhibitor-treated mice developed anemia, as evidenced by a rapid decline in peripheral red blood cell (RBC) parameters, hemoglobin (HGB) levels, and hematocrit (HCT) were significantly reduced following PLK1 inhibitor administration, whereas white blood cell (WBC) counts, mean corpuscular volume (MCV), and mean corpuscular hemoglobin (MCH) remained unaffected (Figure 4B). Upon skeletal dissection of the lower limbs, the BM cell suspension of PLK1 inhibitor -treated mice appeared markedly paler compared to control mice, a visual difference corroborated by the reduced BM cell counts (Figure 4C). Additionally, severe splenomegaly was observed in the PLK1 inhibitor-treated group, and flow cytometry analysis revealed a significant increase in erythroblast populations in the spleen (Figures 4D–F). In conclusion, PLK1-inhibitor treated mice exhibit pronounced anemia and splenomegaly, accompanied by extramedullary erythropoiesis.

### PLK1 inhibition disrupts hematopoietic stem and progenitor cells homeostasis in mice bone marrow

**FIGURE 5:**
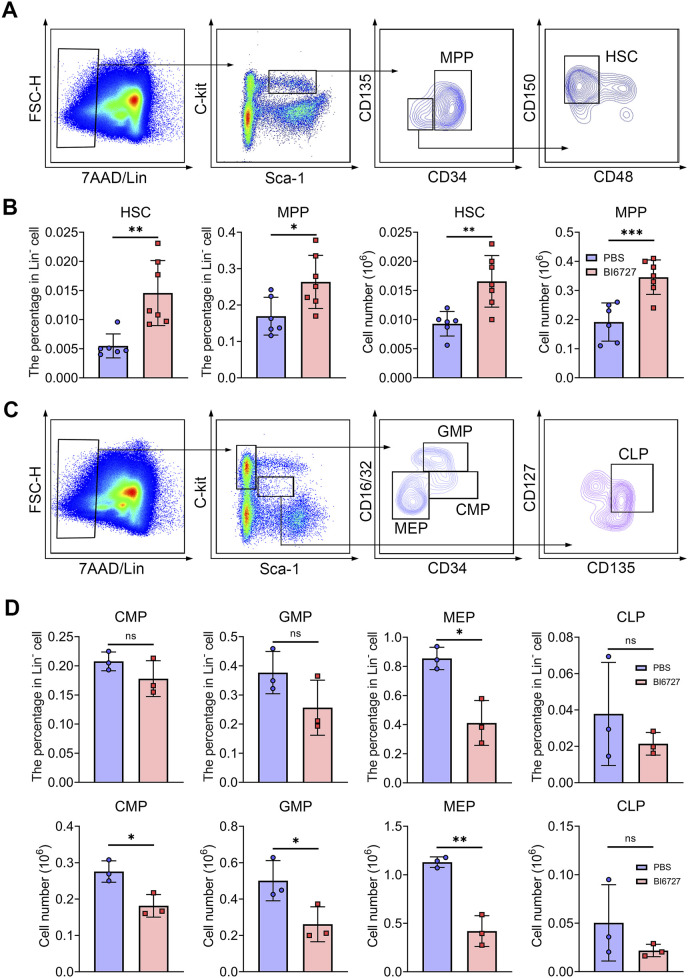
PLK1 inhibition disrupts the maintenance and development of HSPCs. **(A)** Representative flow cytometry plots illustrating the analysis strategy for the LSK^+^ population in mice BM. LSK^+^: Lin^−^Scal1^+^c-Kit^+^. **(B)** Percentage and absolute numbers of HSCs and MPPs in theBM of mice treated with PBS or BI6727. **(C)** Representative flow cytometry plots illustrating the analysis strategy for the LSK^−^ population in mice BM. LSK^−^: Lin^−^Scal1^−^c-Kit^+^. **(D)** The percentage and absolute numbers of CMPs, GMPs, MEPs, and CLPs in BM. N = 3–7. *P < 0.05, **P < 0.01, ***P < 0.001. N.S.: no statistic different.

Hematopoietic stem cells (HSCs) follow a meticulously regulated differentiation pathway, progressing into multipotent progenitors (MPPs). These MPPs then branch into common myeloid progenitors (CMPs), which further differentiate into megakaryocyte-erythrocyte progenitors (MEPs), granulocyte-monocyte progenitors (GMPs), and common lymphoid progenitors (CLPs), demonstrating the complex hierarchical structure of hematopoiesis. To explore whether the severe anemia observed in PLK1 inhibitor-treated mice stemmed from disruptions in HSC homeostasis or the committed differentiation of hematopoietic progenitors, we examined the impact of PLK1 on both HSCs and progenitor cell function. In our flow cytometry gating strategy, the LSK^+^ population was defined as lineage-negative (Lin^−^), Sca-1^+^, and c-Kit^+^, which represents HSCs (LSK^+^CD34^−^CD135^−^CD48^−^CD150^+^) and MPPs (LSK^+^CD34^+^CD135^-^). Conversely, the LSK^−^ population, also lineage-negative, lacked Sca-1, defined as Lin-Sca-1^−^c-Kit^+^, which represents CMP (LSK^−^CD34^+^CD16/32^-^), MEP (LSK^−^CD34^−^CD16/32^-^) and GMP (LSK^−^CD34^+^CD16/32^+^). CLP was defined as LSK^med^CD135^+^CD127^+^. Flow cytometry analysis of BM HSCs and progenitor populations revealed an increased percentage and number of HSCs and MPPs (Figures 5A, B), accompanied by a significant reduction in CMPs, GMPs, and MEPs compared to controls (Figures 5C, D). This indicates that PLK1 inhibitor treatment disrupts normal HSPC homeostasis within the BM. We further assessed the abundance of various terminal myeloid cells. Interestingly, following PLK1 inhibitor treatment, the percentage and number of neutrophils, granulocytes, macrophages, megakaryocytes, T cells, and B cells remained stable (Supplementary Figures S1A–H). These results suggest that while PLK1 inhibitor disrupts early hematopoietic progenitor cell homeostasis, it does not significantly affect the terminal differentiation or maintenance of mature myeloid and lymphoid cell populations.

### PLK1 inhibition leads to defective bone marrow erythroid commitment and differentiation

**FIGURE 6:**
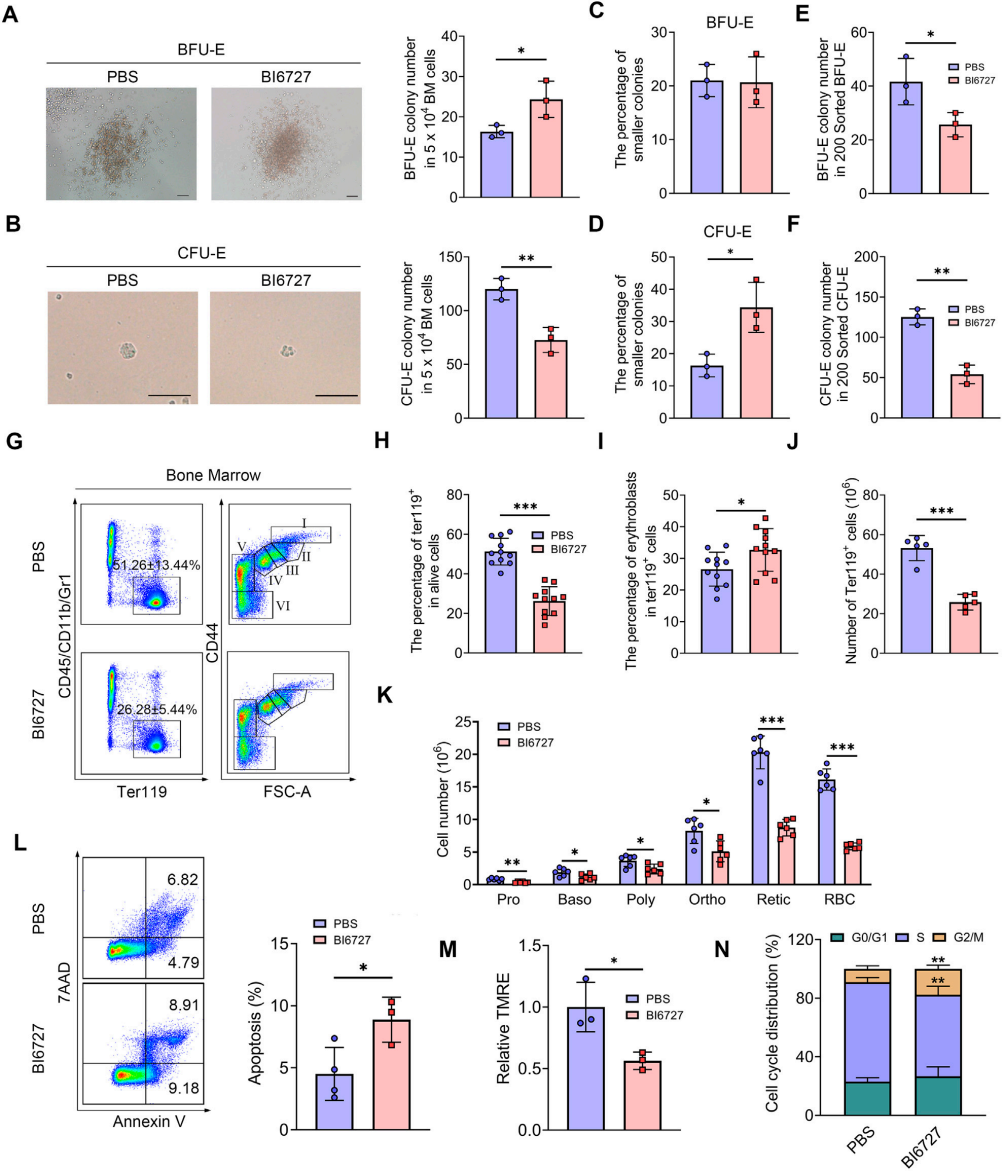
PLK1 inhibition impairs mice erythroid cells differentiation. **(A, B)** Representative images of BFU-E and CFU-E colonies in mice. **(C, D)** Quantitative analysis of the number and size of BFU-E and CFU-E colonies formed by plated BM cells from BI6727-treated and control mice. **(E, F)** BFU-E and CFU-E colonies formed from sorted BFU-E and CFU-E cells. **(G)** Representative flow cytometry plots of BM erythropoiesis in mice. I: Pro; II: Baso; III: Poly; IV: Ortho; V: Retic; VI: RBC. **(H, I)** Percentage and absolute number of BM Ter119^+^ cells. **(J, K)** The percentage and absolute number of BM erythroblasts. **(L)** Flow cytometric gating strategy for identifying apoptotic BM erythroid cells using 7AAD and Annexin V. Quantitative analysis of apoptosis in Ter119^+^ BM cells. **(M)** Assessment of MMP in erythrocytes. **(N)** Quantitative analysis of cell cycle distribution in BM erythroid precursors. N = 3–11. *P < 0.05, **P < 0.01, ***P < 0.001.

We next investigated the influence of PLK1 on erythroid progenitors. Colony-forming unit assays were performed using total BM cells cultured in semisolid medium. Notably, a significant increase in BFU-E colonies was observed, while CFU-E colonies were markedly reduced following PLK1 inhibition (Figures 6A, B). These results indicate that PLK1 deficiency leads to an increase in BFU-E number, accompanied by a reduction in CFU-E number. Additionally, we found that after PLK1 inhibition, the morphology of CFU-E clones became significantly smaller, while no noticeable difference was observed in BFU-E (Figures 6C, D). To further explore these findings, we recently developed a flow cytometry-based method to isolate mouse BM BFU-E and CFU-E cells, defined as Lin⁻c-Kit⁺CD71⁻ and Lin⁻c-Kit⁺CD71^hi^, respectively (Han et al., 2024). Using this method, we sorted BFU-E and CFU-E cells from the BM of both control and PLK1-inhibited mice to assess their colony-forming capacities and found that a reduction in the number of colonies formed by PLK1-inhibited BFU-E and CFU-E cells compared to control (Figures 6E, F). These findings suggest that the colony-forming capacity of PLK1-deficient erythroid progenitors was impaired. In parallel, we analyzed the terminal stages of erythroid differentiation by flow cytometry using the expression of Ter119 and CD44 as markers. The results demonstrated that the percentage and number of Ter119^+^ cells in PLK1-inhibited mice were significantly lower than in control mice, indicating a disruption in erythroid progenitor transformation within the BM. Following PLK1 inhibition, the proportion of erythroblasts was observed to increase, while the number of cells at terminal differentiation stages was decreased (Figures 6G–K).

To investigate whether apoptosis contributes to the observed impairments in erythropoiesis, we conducted flow cytometry analysis using 7AAD and Annexin V staining. Notably, administration of BI6727 increased apoptosis in erythroblasts compared to the control group (Figure 6L). Consistent with the elevated apoptosis, we also detected a reduction in mitochondrial membrane potential (MMP) during erythroid differentiation following PLK1 inhibition (Figure 6M). Moreover, a distinct arrest of the cell cycle at the G2/M phase was observed in BI6727-treated erythroblasts (Figure 6N). These findings suggest that the decrease in erythroid cell numbers is primarily driven by heightened apoptosis and cell cycle arrest resulting from PLK1 inhibition.

## Discussion

This study provides the comprehensive insight into the role of PLK1 in erythroid development, revealing its critical function in regulating erythropoiesis proliferation, differentiation, and survival. Our findings demonstrate that PLK1 inhibition leads to suppressed proliferation, increased apoptosis, and impaired early differentiation of erythroid cells, which subsequently hinders terminal differentiation and enucleation. *In vivo*, PLK1 inhibition resulted in compensatory increases in HSCs and MPPs, while significantly reducing CMPs and MEPs, further disrupting erythroid lineage progression. Mechanistically, PLK1 deficiency primarily induces cell cycle arrest at the G2/M phase, triggering apoptosis and contributing to anemia. These novel findings not only clarify the effects of PLK1 inhibition on erythropoiesis but also provide important theoretical support for understanding anemia as a side effect of PLK1 inhibitor therapy in clinical settings.

Although PLK1-targeted therapies are widely used in clinical trials for cancer treatment, anemia remains a common complication. Multiple preclinical studies on PLK1 inhibitors have shown that different inhibitors lead to significant anemia (Van den Bossche et al., 2016). In this study, we explored the mechanisms of PLK1 inhibitor-induced anemia using Volasertib (BI6727) and GSK461364, two well-known clinical PLK1 inhibitors (Fernández-Sainz et al., 2023; Van den Bossche et al., 2016). Among the commonly used PLK1 inhibitors, BI6727 has been recognized by the FDA as an innovative therapy for leukemia. As a low-molecular-weight, ATP-competitive kinase inhibitor, BI6727 effectively inhibits PLK1, PLK2, and PLK3 and has shown promising effects in various xenograft models and patients with acute myeloid leukemia (AML) (Gjertsen and Schöffski, 2015). However, it is associated with potential side effects. Recent Phase I clinical trials on BI6727 reported that 20% of patients experienced anemia, and other adverse effects included hematological disorders, thrombocytopenia, and neutropenia (Schöffski et al., 2012). GSK461364, another ATP-competitive and selective PLK1 inhibitor, demonstrated antiproliferative activity across multiple tumor cell lines, though it also caused anemia and other blood disorders in clinical trials (Chou et al., 2016).

In this study, we systematically investigated the role of PLK1 in erythroid development by adding two PLK1 inhibitors on day 2 of erythroid cell culture. The results demonstrated that both inhibitors significantly suppressed erythroid cell proliferation. In the early differentiation stage, the inhibitors blocked erythroid progenitors at the BFU-E stage, preventing their transition to CFU-E. We found that GPA expression does not decrease linearly with increasing PLK1 inhibitor concentration, likely due to complex cellular mechanisms such as feedback regulation and compensatory responses beyond PLK1 inhibition. PLK1 has been extensively studied for its role in cell cycle regulation, particularly its involvement in mitotic entry and progression (Jung et al., 2021; Kumar et al., 2017). Numerous studies have explored the effects of PLK1 inhibition on various cell types, including its impact on both proliferation and differentiation (Chiappa et al., 2022; Li et al., 2023). It demonstrated that PLK1 inhibition causes G2/M arrest and leads to apoptosis in various cancer cell lines (Conti et al., 2024; Moolmuang et al., 2024). PLK1 plays a role in the early differentiation of myeloid cells by promoting progenitor maturation (Tao et al., 2017). Our study corroborates these findings by showing that PLK1 inhibition significantly suppresses erythroid cell proliferation, further reinforcing the view that PLK1 is indispensable for cell cycle progression across different cell types.

Specific to erythroid development, very few studies have investigated the role of PLK1 in enucleation. However, our study provides novel insights by demonstrating that PLK1 inhibition significantly reduces enucleation efficiency and increases nuclear abnormalities, which is crucial for matured red blood cell formation. We hypothesize that PLK1 inhibition impairs erythrocyte enucleation by disrupting the proper regulation of the cell cycle and cytoskeletal dynamics essential for nuclear extrusion. PLK1 inhibition leads to erythrocyte nuclear abnormalities may by disrupting the coordination of mitotic spindle formation and chromatin condensation. PLK1 is known to play a critical role in orchestrating the G2/M transition, and its inhibition may lead to cell cycle arrest at this phase (Mouery et al., 2024), preventing the necessary cellular machinery from advancing to the final stages of enucleation. Additionally, PLK1 is involved in the regulation of microtubules and actin filaments (Schmucker and Sumara, 2014), both of which are crucial for the physical process of nucleus expulsion in erythroblasts. By disrupting these cytoskeletal elements, PLK1 inhibition may hinder the mechanical force required for enucleation, ultimately leading to the accumulation of immature erythroid cells unable to complete terminal differentiation.

Our study provides crucial insights into the effects of PLK1 inhibition on the hematopoietic system, demonstrating that BI6727 significantly increases the numbers of HSCs and MPPs in BM. This observation suggests a compensatory response to impaired hematopoiesis, as HSCs typically remain quiescent but proliferate and differentiate when hematopoietic damage occurs. Inhibition of PLK1, such as through BI6727, cause a compensatory increase in HSCs and MPPs, likely in response to disrupted hematopoiesis. This compensatory mechanism, however, is accompanied by a significant reduction in CMPs and MEPs, specifically impairing erythroid lineage development. When PLK1 is inhibited, HSCs and MPPs are unable to efficiently progress through the cell cycle and differentiate into downstream lineages, resulting in their accumulation. Meanwhile, the impaired differentiation process causes a reduction in the number of CMPs, MEPs, and other committed progenitor cells.

Our study demonstrates that PLK1 inhibition blocks erythroid progenitors at the BFU-E stage, preventing progression to CFU-E and disrupting terminal erythroid differentiation, resulting in reduced BM erythrocytes and compensatory splenic erythropoiesis. Mechanistically, PLK1 inhibition has been reported to cause cell cycle arrest at the G2/M phase, inducing apoptosis (Chiappa et al., 2022), which may hinders proper erythroid development. Studies on PLK1 have shown its significant role in regulating apoptosis, particularly in its involvement in both extrinsic and intrinsic apoptotic pathways (Feng et al., 2023; Liao et al., 2022; Luo et al., 2021). PLK1 is essential for proper mitotic progression, and its inhibition has been linked to inducing apoptosis through cell cycle arrest. This arrest triggers apoptotic cascades due to the failure to resolve DNA damage and mitotic defects (Jung et al., 2021). Apoptosis occurs via two main pathways. In the extrinsic pathway, FAS binds to FAS-L, activating a complex with FADD and Caspase 8, which in turn activates Caspases three and seven to initiate cell death (Yanumula and Cusick, 2024; Yuan and Ofengeim, 2024). The intrinsic pathway involves the release of cytochrome c from the mitochondria, which binds to Apaf-1 and Caspase 9, forming an apoptosome and activating the caspase cascade (Jan and Chaudhry, 2019). Therefore, the precise influence of PLK1 inhibitors on these pathways still requires further investigation.

In summary, our research systematically revealed the critical regulatory role of PLK1 in erythroid development, demonstrating that its inhibitors induce anemia by affecting the proliferation, differentiation, enucleation, apoptosis, and cell cycle of erythroid precursor cells. These findings provide new insights and a theoretical basis for the prevention and treatment of PLK1 inhibitor-related anemia, as well as for the development of molecular targeted therapies for cancer.

## Data Availability

The original contributions presented in the study are included in the article/Supplementary Material, further inquiries can be directed to the corresponding authors
