# Supplementary figures and images for: PLK1 inhibition impairs erythroid differentiation
Source: Front Cell Dev Biol. 2024 Dec 23;12:1516704. doi: 10.3389/fcell.2024.1516704 (PMC11701054; doi:10.3389/fcell.2024.1516704)

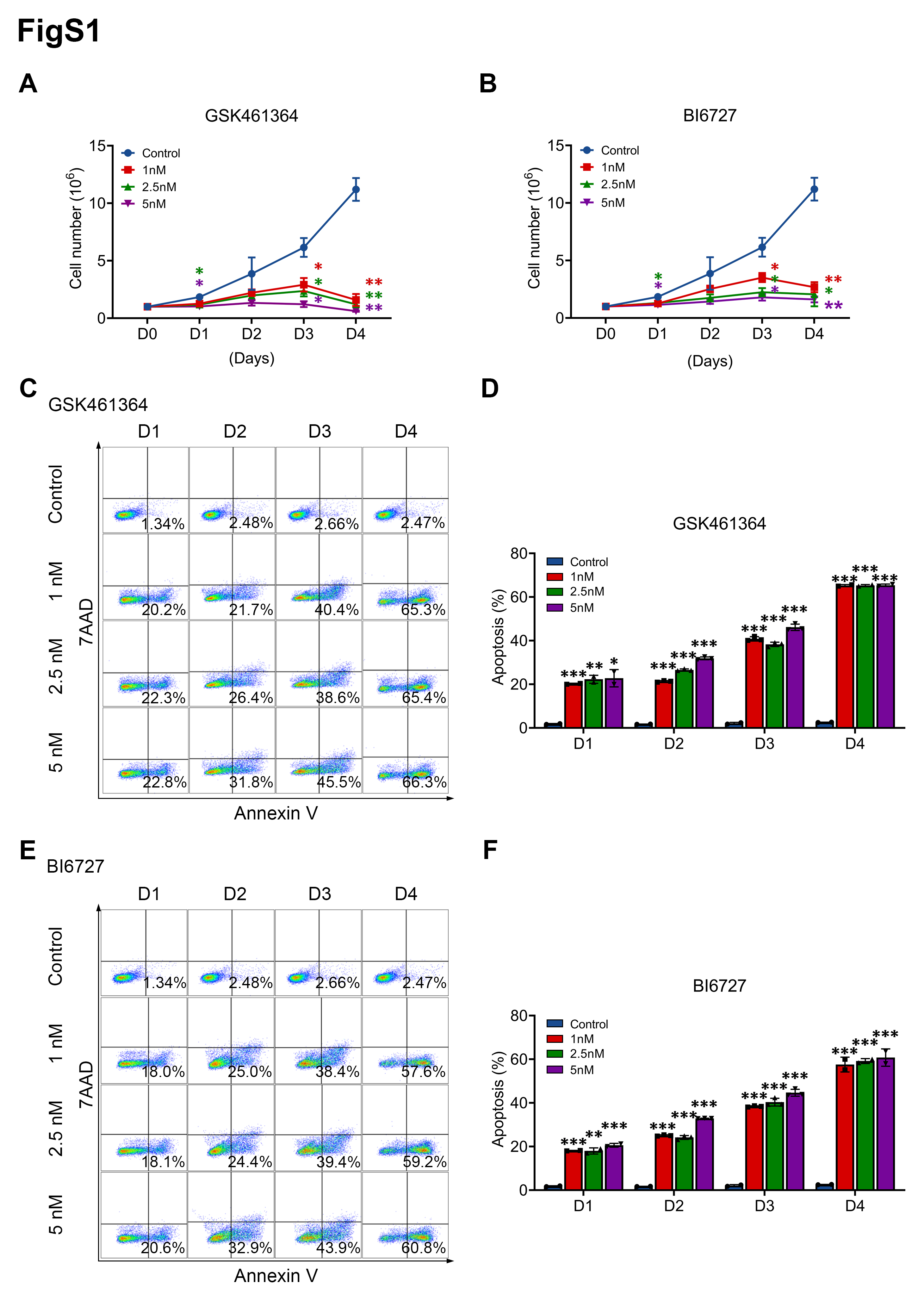

Supplement: Supplementary file 1 [file Image1.jpeg]

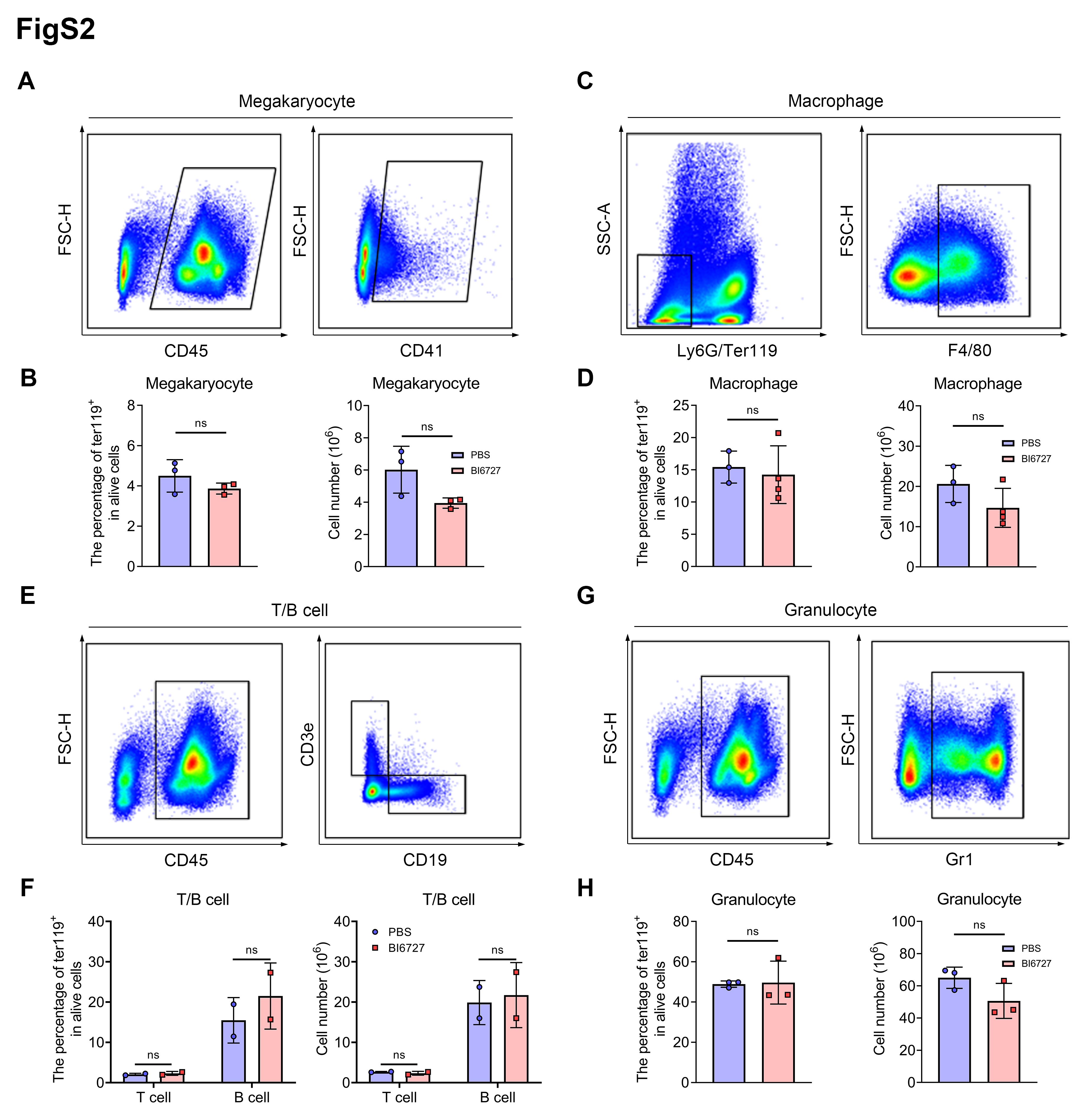

Supplement: Supplementary file 2 [file Image2.jpeg]
